# Multiple sclerosis patients have a distinct gut microbiota compared to healthy controls

**DOI:** 10.1038/srep28484

**Published:** 2016-06-27

**Authors:** Jun Chen, Nicholas Chia, Krishna R. Kalari, Janet Z. Yao, Martina Novotna, M. Mateo Paz Soldan, David H. Luckey, Eric V. Marietta, Patricio R. Jeraldo, Xianfeng Chen, Brian G. Weinshenker, Moses Rodriguez, Orhun H. Kantarci, Heidi Nelson, Joseph A. Murray, Ashutosh K. Mangalam

**Affiliations:** 1 Division of Biomedical Statistics and Informatics–Department of Health Sciences Research Mayo Clinic, 200 1st ST SW, Rochester, MN -55905, USA; 2 Department of Surgical Research Mayo Clinic, 200 1st ST SW, Rochester, MN -55905, USA; 3 Department of Biophysics Mayo Clinic, 200 1st ST SW, Rochester, MN -55905, USA; 4 Mayo Clinic Center for Multiple Sclerosis and CNS Demyelinating Diseases, Department of Neurology, Mayo Clinic College of Medicine, 200 1st ST SW, Rochester, MN-55905, USA; 5 International Clinical Research Center, St. Anne’s University Hospital Brno, Pekařská 53, 656 91 Brno, Czech Republic; 6 Department of Immunology Mayo Clinic, 200 1st ST SW, Rochester, MN -55905, USA; 7 Department of Gastroenterology, Mayo Clinic, Rochester, MN-55905, USA; 8 Department of Pathology, 25 S Grand Ave, 1080-ML, University of Iowa, Iowa City, IA-52242, USA

## Abstract

Multiple sclerosis (MS) is an immune-mediated disease, the etiology of which involves both genetic and environmental factors. The exact nature of the environmental factors responsible for predisposition to MS remains elusive; however, it’s hypothesized that gastrointestinal microbiota might play an important role in pathogenesis of MS. Therefore, this study was designed to investigate whether gut microbiota are altered in MS by comparing the fecal microbiota in relapsing remitting MS (RRMS) (n = 31) patients to that of age- and gender-matched healthy controls (n = 36). Phylotype profiles of the gut microbial populations were generated using hypervariable tag sequencing of the V3–V5 region of the 16S ribosomal RNA gene. Detailed fecal microbiome analyses revealed that MS patients had distinct microbial community profile compared to healthy controls. We observed an increased abundance of *Psuedomonas, Mycoplana, Haemophilus, Blautia*, and *Dorea* genera in MS patients, whereas control group showed increased abundance of *Parabacteroides, Adlercreutzia and Prevotella genera.* Thus our study is consistent with the hypothesis that MS patients have gut microbial dysbiosis and further study is needed to better understand their role in the etiopathogenesis of MS.

Multiple sclerosis (MS) is a pro-inflammatory demyelinating disease of the central nervous system (CNS)^1^. Most MS patients (~85%) present with a relapsing-remitting MS (RRMS) disease course characterized by clearly defined attacks or relapses, followed by a variable degree of recovery. The etiology of MS is complex and poorly understood. Both genetic and environmental factors play a role^2^,^3^, and recent evidence suggests that gut microbiota is one of the key environmental factors. According to the “hygiene hypothesis”, reduced exposure to infections in childhood may increase the risk of allergic and autoimmune diseases^4^,^5^. Supporting this argument, Western societies report an increased incidence of diseases with an autoimmune/allergic component, including MS. Increased constipation and fecal incontinence^6^ and increased gut permeability^7^ in MS patients, and increased occurrence of inflammatory bowel diseases (IBD) in MS patients and their families^8^,^9^, suggest an important gut–CNS connection. Interestingly, gut bacteria can also influence the blood brain barrier integrity^10^. These studies implicate that gut microbiota may potentially be operational in predisposition to or modification of the disease course of MS. Therefore we hypothesized that RRMS patients have gut microbial dysbiosis compared to healthy controls.

To test our hypothesis, we analyze the fecal microbiota composition in patients in the active or remission phases of RRMS and compare it to that of healthy controls. We show that RRMS patients have a distinct microbial community profile compared to healthy controls.

## Results

### Gut Microbiota of RRMS Patients Differs from Healthy controls

**Figure 1:**
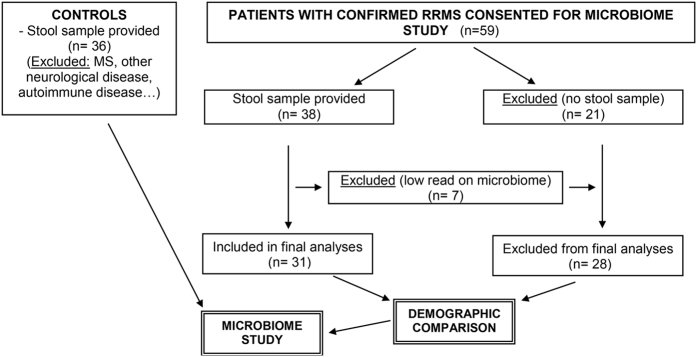
Flowchart explaining RRMS patients and controls recruited for the study with exclusion and inclusion criteria.

**Figure 2:**
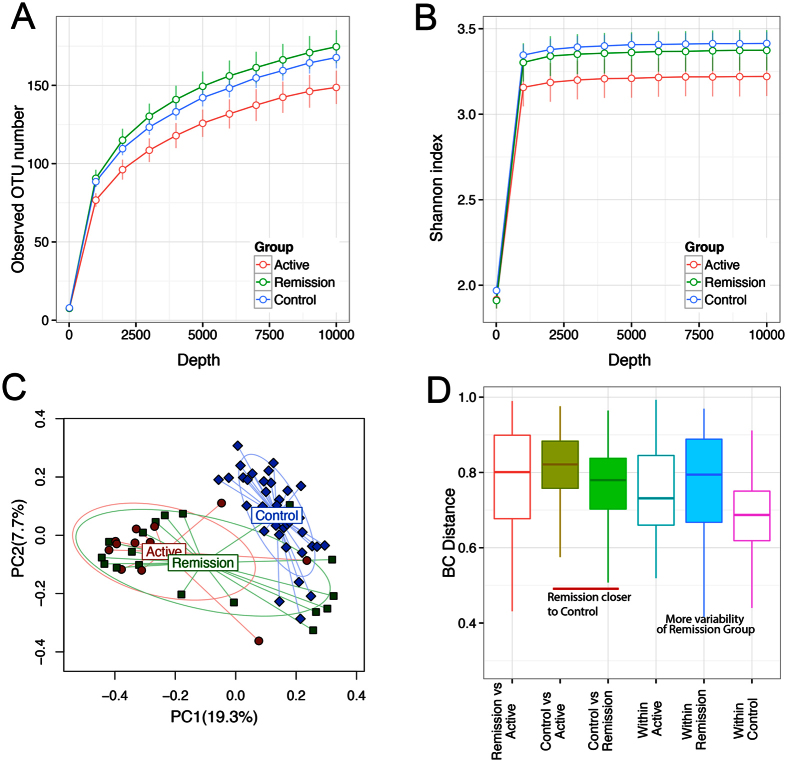
Gut microbiota of MS patients differs from healthy controls. (**A**,**B**) Rarefaction curves comparing the species richness (observed OTU number) and overall diversity (Shannon index) between controls, remission RRMS and active RRMS. The microbiota of active disease has lower diversity. (**C**) Principal coordinate plot based on Bray-Curtis distance matrix. The first two coordinates are plotted with the percentage of variability explained indicated on the axis. Each point represents a sample with colors representing different states. The control samples are significantly different from MS samples. The remission phase samples show greater heterogeneity with subsets resembling control or active disease samples. The ellipses do not represent any statistical significance but rather serve a visual guide to group differences. (**D**) Boxplots comparing Bray-Curtis distances between various groups. The three horizontal lines of the box represent the first, second (median) and third quartiles respectively with the whisk extending to 1.5 inter-quartile range (IQR).

To determine if MS is associated with a change in microbial diversity, fecal samples from 31 RRMS patients were sequenced (Table 1 and Fig. 1) and analyzed using IM_TORNEDO^11^. The median sequencing length obtained was 58,272 bases (range 2,658–894,587). After removing singletons, these sequences were clustered into OTUs based on 97% sequence similarity. Taxonomic classification revealed a typical Western diet diversity profile comprising Firmicutes (58.6%), Bacteroidetes (40.4%), Proteobacteria (0.7%), Actinobacteria (0.1%), and a tail of rare bacterial phyla (0.2%) (Supplemental Figure S1). The overall species richness of the gut microbiota of the RRMS patients was not significantly different from that of the healthy controls (*P* = 0.73 for observed OTU number, Supplemental Figure S2). However, when RRMS patients were divided into patients with active disease and those in remission, there was a trend towards lower species richness in patients with active disease compared to healthy controls (*P* = 0.1). The microbiota in patients in the remission phase exhibited species richness similar to the healthy controls (Fig. 2A). The Shannon diversity index, which considers both the species richness and evenness, also identified a similar decreasing trend in species richness for patients in the active disease state (*P* = 0.2, Fig. 2B).

Bray-Curtis distance-based community analysis revealed that the microbiota structure differed significantly between RRMS patients in both remission and active state and healthy controls (*P* < 0.001, PERMANOVA test, Fig. 2C). Interestingly, the structure of the active state microbiota was also different from that of the remission state (*P* = 0.05, PERMANOVA, Fig. 1D), with the remission state microbiota being more similar to that of the healthy controls (*P* = 0.06, 1000 permutations; Fig. 2D). The remission group appeared to be composed of two subgroups, one that was similar to the active state, and one that was similar to the healthy controls (data not shown). The heterogeneity of the remission group resulted in more variability in the entire RRMS group, as well as in the remission group, compared to the control group as revealed by a distance-based homogeneity test (*P* < 0.001, PERMDISP test; Fig. 2D). We did not observe any effect of treatment status, smoking, or vitamin D supplementation on composition of microbiota (data not shown).

### Abundance of Certain Bacteria Is Associated with RRMS

**Figure 3:**
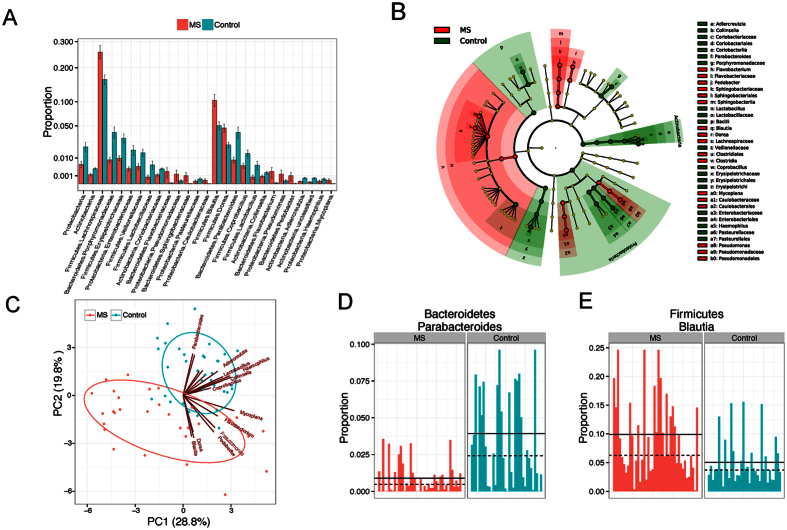
Microbial signatures of the gut microbiota of MS patients. (**A**) Barplots comparing the abundances of differentially abundant taxa between MS and control. These “signature” taxa are selected by Wilcoxon rank-sum tests and a false discovery rate of 5%. Error bars represent standard errors. Phylum, family and genus-level taxa are plotted. (**B**) The “signature” taxa are highlighted on the phylogenetic tree (cladogram) using GraPhlAn with red and green color indicating increase and decreases of abundance in the MS patients. (**C**) Biplots based on principal component analysis of the abundances of the “signature” genera. The first two components are plotted with the percentage of variability explained indicated. Each point represents a sample. The contribution of each genus to the principal components is indicated by the angle and length of the blue line. (**D**,**E)** Barplots comparing the relative abundances of *Parabacteroides* and *Blautia* between MS and control. Each bar represents the relative abundance of a sample.

We used a conservative Wilcoxon rank-sum test to perform differential abundance analyses at the phylum, family, and genus levels, confining the analyses to the taxa with prevalence >10% and maximum proportion >0.002. At a false discovery rate of 5%, we identified 35 differentially abundant taxa (Fig. 3A, Table 2). Within the phylum Actinobacteria, the genera *Adlercreutzia* and *Collinsella* were less abundant in RRMS patients compared to controls. Among Bacteroidetes, *Pedobacter* and *Flavobacterium* genera showed higher abundance, whereas *Parabacteroides* showed lower abundance in MS patients compared to healthy controls. Certain Firmicutes genera such as *Blautia* and *Dorea* were enriched in RRMS patients, whereas others belonging to families Erysipelotrichaceae, Lachnospiraceae, Veillonellaceae and genera *Lactobacillus* and *Coprobacillus* showed lower abundance in RRMS patients compared to healthy controls. Among Proteobacteria, *Pseudomonas* and *Mycoplana* were more abundant in RRMS patients, whereas *Haemophilus* was more abundant in healthy controls (Fig. 3B). Using the relative abundance of these differential abundant taxa, we could separate the RRMS microbiota from the control microbiota (Fig. 3C).

Eleven taxa (Table 2, highlighted in bold) remained significant even when stringent Bonferroni correction (*P-*value cutoff = 0.0005) was used for addressing multiple testing. Using a two log fold changes as a cut-off, we observed that RRMS patients showed enrichment of *Pseudomonas* (Proteobactreia), *Pedobacter* (Bacteroidetes), *Blautia* (Firmicutes), *Dorea* (Firmicutes), and *Mycoplana* (Proteobactreia); whereas healthy controls showed enrichment of *Adlercreutzia* (Actinobacteria), *Collinsella* (Actinobacteria), *Lactobacillus* (Firmicutes) and *Parabacteroides* (Bacteriodetes). Figure 3D shows the abundance data from representative gut microbes increased in control (*Parabacteroides*) or RRMS patients (*Blautia*). Thus, these data indicate that RRMS patients had differential abundance of certain gut microbiota compared to controls.

### Predictive Model Using RF

**Figure 4:**
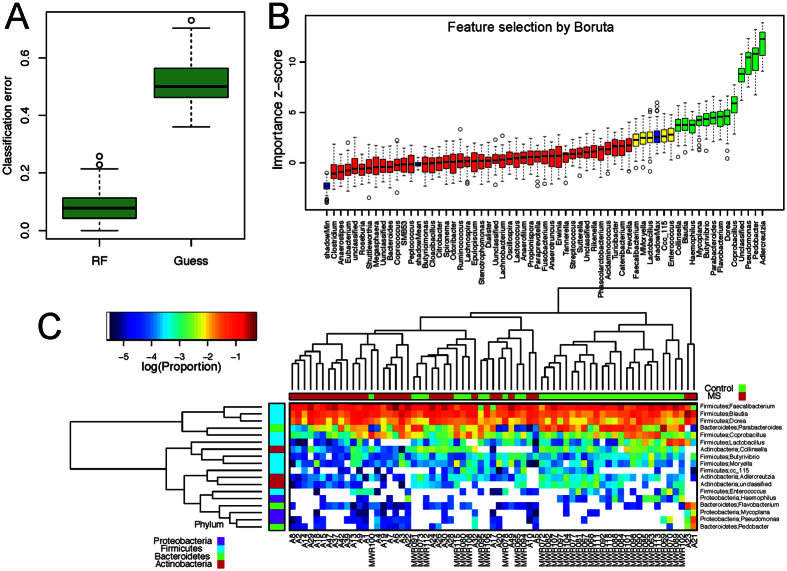
Predictive model based on the genus-level abundance profile using Random Forests (RF). (**A**) Comparison of the classification error of the RF trained model to guess, which always predicts the class label based on the majority class in the training data set. The boxplots are based on the results from 500 bootstrap samples. The three horizontal lines of the box represent the first, second (median) and third quartiles respectively with the whisk extending to 1.5 inter-quartile range (IQR). RF achieves significantly lower classification error. (**B**) Predictive power of individual genera assessed by Boruta feature selection algorithm. Blue boxplots correspond to minimal, average and maximum Z score of shadow genera, which are shuffled version of real genera introduced to RF classifier and act as benchmarks to detect truly predictive genera. Red, yellow and green colors represent rejected, suggestive and confirmed genera by Boruta Selection. (**C**) Heatmap based on the abundance Boruta selected genera. Hierarchical clustering (Euclidean distance, complete linkage) shows that MS samples tend to cluster together.

We next assessed the predictive power of the gut microbiota using RF, which predicts disease status based on an ensemble of decision trees. We used RF to build a predictive model based on the gut microbiota profile using the genus-level abundance data as the input. Based on 500 bootstrap samples, we achieved a mean classification error of 0.08, compared to 0.49 based on random guess, meaning we always predicted the class label (RRMS status) of the test sample to be the class label of the majority class seen in the training set (*P* < 2.2 × 10^−16^, Friedman Rank Sum test, Fig. 4A). RRMS samples differ substantially in genus-level abundance from the control samples. The relative importance of each genus in the predictive model was assessed using mean decreasing accuracy and Gini coefficient. We used the Boruta algorithm to select significant genera, and 18 genera were confirmed for their importance in prediction (Fig. 4B). This prediction model confirmed that certain genera such as *Adlercreutzia, Pedobacter, Pseudomonas, Coprobacillus, Dorea, Flavobacterium, Parabacteroides, Mycoplana, Haemophilus, Blautia*, and *Collinsella* were predictive of the disease state. Identification of the same genera in the earlier abundance analysis (Fig. 3A) indicated the robustness of both analyses. Hierarchical clustering based on the abundance profile of these confirmed genera showed that the MS samples generally clustered together (Fig. 4C).

**Figure 5:**
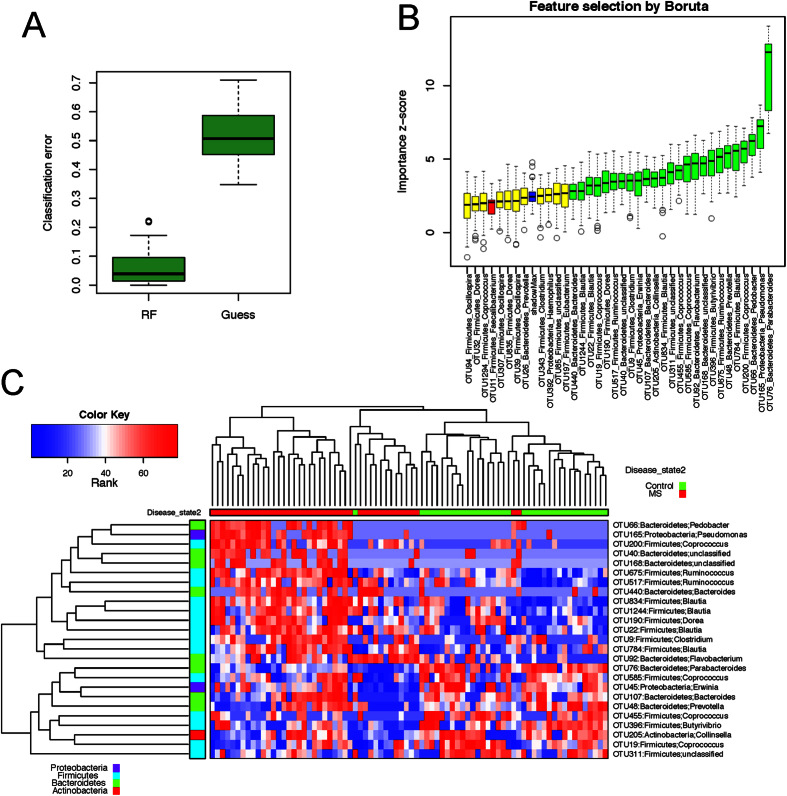
Predictive model based on the OTU level abundance profile using Random Forests (RF). (**A**) Comparison of the classification error of the RF trained model to guess, which always predicts the class label based on the majority class in the training data set. The boxplots are based on the results from 500 bootstrap samples. The three horizontal lines of the box represent the first, second (median) and third quartile respectively with the whisk extending to 1.5 inter-quartile range (IQR). RF achieves significantly lower classification error. (**B**) Predictive power of individual genera assessed by Boruta feature selection algorithm. Blue boxplots correspond to minimal, average and maximum Z score of shadow genera, which are shuffled version of real genera introduced to RF classifier and act as benchmarks to detect truly predictive genera. Red, yellow and green colors represent rejected, suggestive and confirmed genera by Boruta Selection. (**C**) Heatmap based on the abundance Boruta selected genera. Hierarchical clustering (Euclidean distance, complete linkage) shows that MS samples tend to cluster together.

We repeated the analysis using the abundance data of 291 OTUs (Fig. 5). Even lower classification error of 0.04 was achieved based on these bootstrap samples. The Boruta algorithm confirmed 25 OTUs, with the majority of them belonging to the same genera identified above (Fig. 5B,C). The new genera identified by the analysis were *Prevotella, Clostridium*, and *Erwina*. OTU belonging to Certain *Prevotella* species was observed more frequently in healthy controls compared to RRMS patients (Supplemental Figure S3). The high concordance between the results obtained from two methods indicated that the RF prediction model was a powerful tool for selecting microbes that were associated with the RRMS.

### Functional Analysis

**Figure 6:**
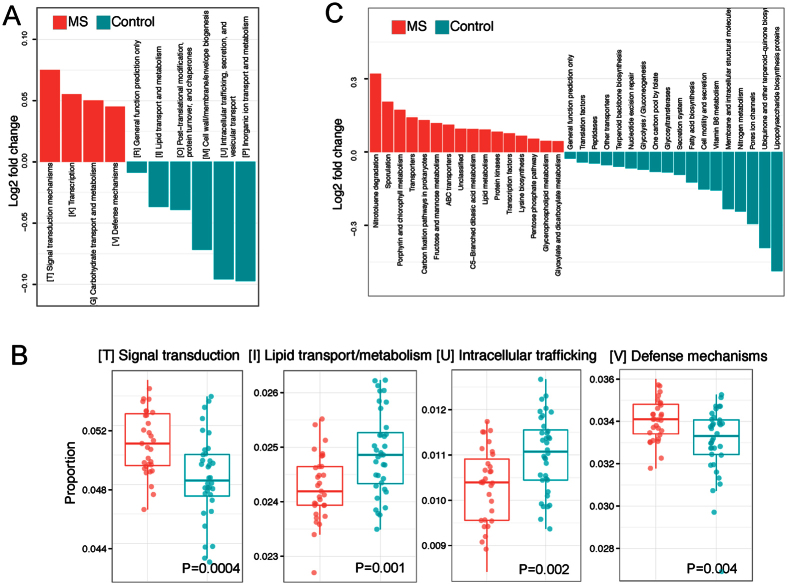
Functional analysis of the MS gut microbiota. PICRUSt was used to infer the functional content of the gut microbiota based on the 16S data. (**A**) Log2 fold change of the abundances of COG categories showing significant difference between MS and control at a false discovery rate of 5%. Red and blue colors indicate increase and decrease in the MS samples. (**B**) Boxplots comparing the abundance distribution of the top 4 differential abundant COG categories. (**C**) Log2 fold change of the abundances of KEGG categories showing significant difference between MS and control at a false discovery rate of 5%.

We used PICRUSt to infer the functional content of the microbiota based on closed-reference OTU picking. Twenty COG functional categories were tested. At an FDR of 0.05, we identified 10 differentially abundant COGs (Fig. 6A), Supplemental Table S4). Four COG categories, including signal transduction mechanism, lipid transport and metabolism, intracellular trafficking, and defense mechanisms, exhibited the most significant differences (Fig. 6B), Supplemental Table S4). In contrast to the taxonomic abundance, the functional abundance exhibited much less variability, with fold changes ranging narrowly between 1% and 6%. However, even under such small effect sizes we were still able to detect differentially abundant COGs because of even smaller within-group variability. We also compared 104 KEGG pathways. At an FDR of 0.05, we identified 34 differentially abundant pathways, indicating diverse change in the functions of the RRMS microbiota compared to controls (Fig. 6C). Three KEGG pathways, fatty acid biosynthesis, glycolysis, porphyrin and chlorophyll metabolism, and transporters survived the Bonferroni correction.

## Discussion

The present study demonstrates that RRMS patients have a distinct fecal microbiome compared to healthy controls, with certain gut microbes showing decreased or increased abundance in RRMS patients compared to controls. The strength of our study lies in the abundance analyses, as well as utilization of prediction models to identify the bacterial taxa differentially expressed in the disease state. A high concordance rate between abundance and the prediction model is an added advantage of our study. Thus, our data points toward an important role of the gut microbiota in the RRMS patients. These results provide clear evidence that a larger independent replication study focusing on the identified findings will be needed in which MS phase-specific outcome analyses can also be conducted.

In our study, analysis of species richness (α-diversity) showed no difference between total RRMS patients and healthy controls; however, RRMS patients with active disease showed decreased species richness compared to patients in remission and controls. Decreased species richness in RRMS patients with active disease, points towards an important role of gut microbiota in disease exacerbation. Future studies analyzing gut microbiota at various time points might help in a better understanding of the role of gut microbiota in disease exacerbation. We also observed a lower abundance of *Bacteroidetes* in RRMS patients, which is in agreement with an earlier study showing significant decrease in *Clostridium* and *Bacteroidetes* species in RRMS patients^12^. In another exploratory study of 15 MS patients, certain taxa such as *Fecalibacterium* were decreased in MS patients compared to healthy controls^13^. However we observed similar abundance of *Fecalibacterium* between RRMS and healthy controls. A number of factors such as different 16s primer sets, sequencing techniques, and geographical location might be responsible for the difference in abundance of *Fecalibacterium* between two studies.

Thus our study is in agreement with earlier reports indicating that MS patients had dysbiosis of fecal microbiota in the gut. We did not observe any effect of factors such as smoking, family history, or treatment status on abundance of microbiota; however, we cannot rule out their effect on gut microbiota due to the small sample size in each group. An increased Firmicutes in RRMS with active disease cannot be attributed to obesity alone as a recent meta-analysis of human gut microbiota associated with IBD and obesity had shown that ratio of Firmicutes:Bacteroidetes is not a consistent feature distinguishing lean from obese human microbiota^14^.

Our study was not biased by specific pre-determined candidate strata based on a previous gut microbiome analyses. We nevertheless identified several organisms—including *Parabacteroides* and *Prevotella* (Bacteroidetes), *Adlercreutzia* and *Collinsella* (Actinobacteria), and *Erysipelotrichaceae* (Firmicutes)—that were decreased in RRMS compared to healthy controls. Interestingly*, Prevotella*, *Parabacteroides*, and *Adlercreutzia* are associated with the metabolism of phytoestrogens and plant-derived xenoestrogen^15^,^16^,^17^, whereas *Parabacterodes* and *Erysipelotrichaceae* are involved in bile acid metabolism. Metabolites derived from the metabolism of phytoestrogens (lignan and isoflavone) and bile acids play an important role in maintaining homeostasis at mucosal surfaces through the induction of anti-inflammatory responses^18^. Low estrogen states such as menopause and the postpartum period are clearly associated with increased activity in women with MS^19^. Further, treatment with estrogens can suppress and/or protect animals from disease in an experimental model of human MS, experimental autoimmune encephalomyelitis (EAE)^20^,^21^. When combined with the fact that RRMS patients have reduced levels of bacteria responsible for metabolizing phytoestrogen, this might implicate regulation of estrogen receptor signaling by gut microbiota and/or their metabolites in the etiopathogenesis of RRMS.

Erysipelotrichaceae and Veillonellaceae families (Firmicutes) showed lower abundance in RRMS cases compared to controls. Erysipelotrichaceae plays an important role in bile acid metabolism^22^, which can induce anti-inflammatory properties^23^. In one report patients with Crohn’s disease had decreased levels of Erysipelotrichaceae in their fecal samples and intestinal biopsies^24^. Veillonellaceae is closely related to Clostridium, a beneficial commensal that has been shown to induce regulatory T cells^25^.

RF OTU analysis indicated a decreased abundance of *Prevotella* in MS patients compared to controls. Decreased *Prevotella* levels have been reported in diseases such as type-1-diabetes and autism^26^,^27^. In a separate study (manuscript under review) from our lab, we have observed that one of the Prevotella species- *Prevotella histicola* can suppress disease in experimental model of MS.

Among Proteobacteria, *Caulobacteraceae, Pseudomonas* and *Mycoplana* were more abundant in MS patients, whereas Enterobacteriales were more abundant in the control group. Previous studies have suggested an association between certain Proteobacteria and the pathogenesis of IBD^28^. Two Firmicutes, *Blautia* and *Dorea*, were more abundant in patients with RRMS compared to controls, however they have been linked both positively^29^ and negatively^30^ with inflammatory diseases. Increased *Pedobacter* in microbiome samples has been attributed to contaminated DNA extraction kit reagents^31^. Future studies on large patients’ cohort as well as metagenomics studies with help in defining the role of gut microbiota which are abundant in MS patients.

The functional analysis (PICRUSt) data demonstrated modulation of pathways involved in fatty acid metabolism, defense mechanisms, and glycolysis, which further strengthens our observations from the abundance and RF analyses. We observed a decrease in the abundance of microbes involved in fatty acid metabolism (bile metabolism) and an increase of pathways involved in the defense mechanisms (induction of anti-inflammatory pathways by phytoestrogen and bile acids metabolites). However, the sequencing method alone does have its limitations, as it does not give direct data on the functionally important changes of the microbiota.

The gut/nutrient-centric nature of human evolution suggests an important role for gut-associated microbiota in maintaining homeostasis and human health. Thus, a perturbation of gut microbiota can result in the depletion of certain microbial communities and important metabolites that can lead to impaired immune homeostasis resulting in predisposition to inflammatory disease such as RRMS. Decreased species richness in MS patients with a recent attack compared to those in remission, and modulation of microbes associated with the induction of anti-inflammatory pathways, highlight the importance of the gut microbiome in the etiology of MS. The abundance of microbes involved in the phytoestrogen metabolic pathway is interesting as there is increased female prevalence in MS patients. Based on our observations, gut microbiota may be one of the missing environmental factors responsible for the precipitation of disease in genetically susceptible individuals. Large studies that include time series of samples and methodology to study the functional changes in the intestinal microbiota are needed for the evaluation of the role of gut microbiota in the modulation of immune system response.

## Methods

### Standard Protocol Approvals, Registrations, and Patient Consents

The study was done in accordance with the guidelines approved by Mayo Clinic Institutional Review Board (IRB). A prior written informed consent was obtained from all the subjects to participate in the study.

### Patients and Samples

RRMS patients (n = 62) between 18 and 80 years of age and fulfilling McDonald diagnostic criteria for MS^32^ with an expanded disability status scale (EDSS) score between 1 and 6 were recruited at the MS Clinic at Mayo Clinic (Rochester, MN). The demographic data for the RRMS patients and healthy controls are summarized in Table 1 and Fig. 1. We also collected data on smoking, vitamin D supplement, and treatment status. Fifty-nine patients with confirmed RRMS consented for microbiome study, 38 of whom provided stool samples. Seven samples were excluded due to low read on sequencing. Thirty-one samples were included for analysis (Fig. 1).

RRMS patients were defined as being in an active disease state if the stool sample was collected within a month of a relapse; otherwise, they were considered to be in remission. Healthy controls (n = 36) included an age- and sex-matched cohort with no known disease symptoms. The general exclusion criteria included prior surgeries, including colectomy, ileectomy, and gastrectomy, and excluded minor procedures, such as cholecystectomy, hemorrhoidectomy, and appendectomy. Any patients or controls currently taking antibiotics or probiotic supplements, or having a known history of disease with an autoimmune component such as MS, rheumatoid arthritis, type-1-diabetes, and IBD, were also excluded from the study.

### Sample Collection, 16S Amplicon Preparation, Sequencing, and Processing

Subjects were provided with Commode Specimen Collection kits (Fisher Scientific Inc., Pittsburgh, PA, USA) to collect stool. If patients were unable to give the stool sample during their stay at Mayo Clinic, then they were instructed to collect the sample at home and send it through overnight FedEx delivery. Samples were frozen at −70 °C within 24 hours of receipt. Microbial DNA was extracted from fecal material of each sample using the MoBio PowerSoil Kit (MoBio Laboratories, Carlsbad, CA, USA) as per the manufacturer’s instruction with a bead-beating step. Sequencing of the V3–V5 region of the 16s rRNA was performed as described previously^11^. The raw 16S data were processed by IM-TORNADO^11^ to form operational taxonomic units (OTUs) at 97% similarity level.

### Statistical Analyses

α-diversity (Observed OTU number and Shannon index) and β-diversity (Bray-Curtis and UniFrac distances) measures were calculated based on the rarefied OTU counts. PERMANOVA was used to test for association between disease status and the overall microbiota composition based on distance matrices (“adonis” function in R package “vegan”). PERMDISP2 was used to test the homogeneity of group dispersions (“betadisper” function in R package “vegan”). Significance was assessed by 1000 permutations. Differential abundance analysis was performed using the Wilcoxon rank-sum test at phylum, family, and genus levels. False discovery rate (FDR) control based on the Benjamini-Hochberg procedure was used to correct for multiple testing. Differentially abundant taxa were highlighted on the tree using the GraPhlAn software (huttenhower.sph.harvard.edu/graphlan).

### Predictive Modeling using Random Forests

Random Forests (RF) was used to predict the disease status based on the microbiota profile (genus-level relative abundance data) using default parameters of the R implementation of the algorithm (R package “randomForest”). Bootstrapping (n = 500) was used to assess the classification accuracy. The classification performance was compared to random guess, where the class label of a test sample was predicted to be the label of the majority class in the training set, and significance of difference was assessed by a Friedman rank-sum test. The Boruta algorithm was used to select the taxa that have predictive power (R package “Boruta”). PICRUSt was used to predict the abundance of functional categories such as clusters of orthologous group (COG) categories and Kyoto Encyclopedia of Genes and Genomes (KEGG) pathways based on the 16S rDNA sequence data^33^. All the statistical analyses were performed in R-3.0.2 (R Development Core Teams).

## Additional Information

**How to cite this article**: Chen, J. *et al*. Multiple sclerosis patients have a distinct gut microbiota compared to healthy controls. *Sci. Rep.* **6**, 28484; doi: 10.1038/srep28484 (2016).

## Supplementary Material

Supplementary Information

## Figures and Tables

**Table 1:** Clinical and demographic features of MS patients and control.

	RRMS			Control
	Active	Remission	Total	
N	12	19	31	36
Sex (M/F)	3/9	7/12	10/21	14/36
Age (SD)	39.3 (10.6)	45.2 (10.2)	42.9 (10.6)	40.3 (7.3)
Age of onset	32.6 (7.9)	37.2 (11.5)	35.4 (10.4)	–
BMI	32.7 (7.4)	26.2 (5.5)	28.0 (6.3)	27.8 (4.7)
Disease severity				–
EDSS				
<3	7	12	19	
3–5	3	0	3	
>6	0	2	2	
ND	3	5	8	
Therapy				
IFNb	7	7	14	
Copax	0	1	1	
Tysabri	1	4	5	
None	4	7	11	

**Table 2:** Differentially abundant taxa between MS and control samples at phylum, family and genus-level.

	P value	Q value	MS Mean	Control Mean	Log2 fold change	MS prevalence	Control prevalence
Phylum*							
	**4.09E-06**	**3.27E-05**	**1.32E-03**	**3.46E-03**	**−1.39**	**31**	**36**
Proteobacteria	3.60E-03	1.44E-02	5.63E-03	2.03E-02	−1.85	29	36
Family*							
**Actinobacteria; Coriobacteriaceae**	**2.13E-06**	**2.49E-05**	**1.22E-03**	**3.34E-03**	**−1.46**	**31**	**36**
Bacteroidetes; Flavobacteriaceae	1.76E-03	7.70E-03	2.26E-03	1.41E-05	7.33	25	14
**Bacteroidetes; Porphyromonadaceae**	**4.85E-04**	**3.40E-03**	**8.60E-03**	**3.95E-02**	**−2.20**	**30**	**36**
**Bacteroidetes; Sphingobacteriaceae**	**6.69E-08**	**2.34E-06**	**1.03E-03**	**1.89E-06**	**9.09**	**21**	**1**
Firmicutes; Erysipelotrichaceae	6.90E-04	3.63E-03	9.77E-03	3.10E-02	−1.67	31	36
Firmicutes; Lachnospiraceae	2.67E-03	1.04E-02	2.58E-01	1.61E-01	0.68	31	36
Firmicutes; Lactobacillaceae	5.38E-03	1.71E-02	7.24E-04	5.30E-03	−2.87	24	33
Firmicutes; Veillonellaceae	7.25E-04	3.63E-03	4.54E-03	1.44E-02	−1.67	31	36
**Proteobacteria; Caulobacteraceae**	**3.58E-04**	**3.13E-03**	**2.03E-04**	**1.38E-06**	**7.20**	**15**	**3**
Proteobacteria; Enterobacteriaceae	9.75E-03	2.84E-02	3.44E-03	1.69E-02	−2.29	27	35
Proteobacteria; Pasteurellaceae	4.35E-03	1.52E-02	1.07E-04	3.67E-04	−1.78	6	18
**Proteobacteria; Pseudomonadaceae**	**6.96E-07**	**1.22E-05**	**1.44E-03**	**1.51E-04**	**3.25**	**23**	**3**
Genus*							
**Actinobacteria; Adlercreutzia**	**1.39E-07**	**4.16E-06**	**8.99E-05**	**5.16E-04**	**−2.52**	**25**	**34**
Actinobacteria; Collinsella	6.87E-03	3.17E-02	8.69E-04	1.87E-03	−1.11	26	30
Actinobacteria; unclassified	1.80E-03	1.34E-02	4.57E-05	4.92E-04	−3.43	19	24
Bacteroidetes; Flavobacterium	1.76E-03	1.34E-02	2.26E-03	1.41E-05	7.33	25	14
Bacteroidetes; Parabacteroides	7.25E-04	7.25E-03	8.51E-03	3.93E-02	−2.21	30	36
**Bacteroidetes; Pedobacter**	**6.69E-08**	**4.01E-06**	**1.03E-03**	**1.89E-06**	**9.09**	**21**	**1**
**Firmicutes; Blautia**	**4.38E-04**	**5.25E-03**	**1.04E-01**	**5.03E-02**	**1.04**	**31**	**36**
Firmicutes; Coprobacillus	2.24E-03	1.34E-02	5.01E-03	1.38E-02	−1.46	31	36
Firmicutes; Dorea	2.05E-03	1.34E-02	4.66E-02	2.26E-02	1.05	31	36
Firmicutes; Lactobacillus	5.81E-03	2.90E-02	7.23E-04	5.24E-03	−2.86	24	33
Proteobacteria; Haemophilus	4.35E-03	2.37E-02	1.07E-04	3.67E-04	−1.78	6	18
**Proteobacteria; Mycoplana**	**3.58E-04**	**5.25E-03**	**2.03E-04**	**1.38E-06**	**7.20**	**15**	**3**
**Proteobacteria; Pseudomonas**	**6.96E-07**	**1.39E-05**	**1.44E-03**	**1.51E-04**	**3.25**	**23**	**3**

^*^-An FDR of 0.05 is used to identify these taxa.
