# Supplementary material for: Multiple sclerosis patients have a distinct gut microbiota compared to healthy controls
Source: Sci Rep. 2016 Jun 27;6:28484. doi: 10.1038/srep28484 (PMC4921909; doi:10.1038/srep28484)
Supplement: Supplementary Information [file srep28484-s1.doc]

**Multiple sclerosis patients have a distinct gut microbiota compared to healthy controls**

Jun Chen, Ph.D.^1*^, Nicholas Chia, Ph.D.^2,3^, Krishna R Kalari, Ph.D.^1^, Janet Z Yao^2^, Martina Novotna, M.D.^4,5^, M Mateo Paz Soldan, M.D., Ph.D.^4^, David H Luckey^6^, Eric V Marietta, Ph.D.^7^, Patricio R Jeraldo, Ph.D.^2^, Xianfeng Chen^1^, Brian G Weinshenker, M.D.^4^, Moses Rodriguez, M.D.^4,6^, Orhun H Kantarci, M.D.^4^, Heidi Nelson, M.D.^2^, Joseph A. Murray, M.D.^6,7^ and Ashutosh K. Mangalam, Ph.D.^8,9^,*

**Supplementary Information**

**Supplemental Figure S1:** **Phylum-level comparison of fecal microbiota in RRMS patients and healthy controls.**  Bar colors depicts average proportion of phyla level abundance in all samples of a particular category. Majority of bacteria belonged to either Firmicutes (Blue color) or Bacteroidetes phyla.

**Supplemental Figure S2:** The overall species richness of microbiota was similar between MS patients and healthy controls. Rarefaction curves comparing the species richness (observed OTU number) between RRMS patients (MS) and controls.

**Supplemental Figure S3**: **Healthy control group had higher relative abundance of *Prevotella* compared to MS patients group.** Bar plot showing relative abundance of an OTU denoting particular *Prevotella* species between control and MS patients. Each bar represents the relative abundance of Prevotella in a sample.

**Supplemental Table S4:** Differentially abundant taxa between MS and control samples at phylum, family and genus-level

| **Pathways** | **P value** | **Q value** | **MS Mean** | **Control**  **Mean** | **log2Fold Change** |
| --- | --- | --- | --- | --- | --- |
| **[G] Carbohydrate transport and metabolism** | 9.86E-03 | 2.45E-02 | 9.96E-02 | 9.62E-02 | 0.050 |
| **[I] Lipid transport and metabolism** | 9.72E-04 | 9.72E-03 | 2.43E-02 | 2.49E-02 | -0.037 |
| **[K] Transcription** | 5.47E-03 | 1.82E-02 | 8.43E-02 | 8.12E-02 | 0.055 |
| **[M] Cell wall/membrane/envelope biogenesis** | 2.18E-02 | 4.35E-02 | 6.97E-02 | 7.33E-02 | -0.072 |
| **[O] Post-translational modification, protein turnover, and chaperones** | 4.27E-03 | 1.71E-02 | 2.99E-02 | 3.07E-02 | -0.039 |
| **[P] Inorganic ion transport and metabolism** | 1.10E-02 | 2.45E-02 | 4.48E-02 | 4.79E-02 | -0.097 |
| **[R] General function prediction only** | 7.53E-03 | 2.15E-02 | 1.11E-01 | 1.12E-01 | -0.009 |
| **[T] Signal transduction mechanisms** | 3.20E-04 | 6.40E-03 | 5.09E-02 | 4.83E-02 | 0.075 |
| **[U] Intracellular trafficking, secretion, and vesicular transport** | 2.05E-03 | 1.36E-02 | 1.03E-02 | 1.10E-02 | -0.096 |
| **[V] Defense mechanisms** | 4.27E-03 | 1.71E-02 | 3.41E-02 | 3.30E-02 | 0.045 |

An FDR of 0.05 is used to identify these categories
